# Angiogenesis activity of *Jatropha curcas* L. latex in cream formulation on wound healing in mice

**DOI:** 10.14202/vetworld.2018.939-943

**Published:** 2018-07-15

**Authors:** Ummu Balqis, Darmawi, Cut Dahlia Iskandar, Muhammad Nur Salim

**Affiliations:** 1 Laboratory of Pathology, Faculty of Veterinary Medicine, Syiah Kuala University, Banda Aceh 23111, Indonesia; 2 Laboratory of Microbiology, Faculty of Veterinary Medicine, Syiah Kuala University, Banda Aceh 23111, Indonesia; 3 Laboratory of Research, Faculty of Veterinary Medicine, Syiah Kuala University, Darussalam, Banda Aceh 23111, Indonesia; 4 Laboratory of Histology, Faculty of Veterinary Medicine, Syiah Kuala University, Banda Aceh 23111, Indonesia; 5 Study Program of Mathematics and Applied Sciences, Syiah Kuala University, Banda Aceh 23111, Indonesia

**Keywords:** angiogenesis, CD34, *Jatropha curcas* latex cream, wound healing

## Abstract

**Aim:**

The aim of this research was to determine the angiogenesis activity of *Jatropha curcas* latex in cream formulation on CD34 immune expression during wound healing phase in mice skin.

**Materials and Methods:**

Amount of 36 2-month-old male mice were used between 30 and 40 g. To surgical procedures, wound skin incision was performed 2.0 cm in length until subcutaneous on the paravertebral of each animal. The treatment was carried under locally anesthetized with procaine cream. All mice were divided into four groups, namely the base cream as control group (A), sulfadiazine 0.1% cream (B), *Jatropha curcas* latex cream 10% (C), and *J. curcas* latex cream 15% (D). All groups were treated entire surface of wound. All experiments were performed twice a day for 10 days. Experiments were terminated on days 3, 7, and 10, respectively. The wound healing was assayed in stained histological section in immunohistochemical of the wounds. The CD34 expression was investigated under a microscope.

**Results:**

The results showed that the cream from 10% and 15% latex *J. curcas* revealed moderate immune reaction to CD34 on days 3 and 7 in wound healing of mice skin.

**Conclusion:**

We concluded that the cream from 10% and 15% latex *J. curcas* has potential as angiogenesis activity in wound healing of mice skin.

## Introduction

The wound is defined as a raw skin surface and lost its surface epithelial coverage caused by various types of injuries or diseases [1,2]. It can be damaged or disruption to the normal anatomical structure and function of skin [3]. Wounds that exhibit impaired healing, including delayed acute wounds and chronic wounds, generally have failed to progress through the normal stages of healing [4]. Neovascularization or angiogenesis is important for wound healing process. Angiogenesis plays a role in wound healing by forming new blood vessels from preexisting vessels by invading the wound clot and organizing into a microvascular network throughout the granulation tissue [5]. However, angiogenesis also plays a role in the provision of nutrients and oxygen to the wound area and increases the formation of granulation tissue with its collagen and connective tissue proteins deposition [6]. A subpopulation of hematopoietic progenitor CD34 cells could directly contribute to the neovascularization and wound repair process [7]. CD34 is a cell surface marker that is expressed by a broad range of cells including hematopoietic, stromal, epithelial, and endothelial cells [8,9].

Wound healing is influenced by many factors including the type of medication used. One is the use of traditional medicine. The use of traditional medicine is increasingly favored because of fewer side effects such as drugs from chemicals [10], the cost is relatively low, and it is easily accessible [11]. Secondary metabolites from herbal plants that are potential as microbial agents may help overcome the problem of antibiotic resistance [12]. *Jatropha curcas* L. (Euphorbiaceae) is multiple purpose with potential for biodiesel production and medicinal uses [13]. Some of the benefits of *J. curcas* are that they can adapt well to dry land, are easy to cultivate, and their utilization does not compete with food crops such as cassava, maize, coconut, and palm oil [14]. *J. curcas* is generally grown as a live fence for protection of agricultural fields from damage by livestock as it is not eaten by cattle [15] and to prevent erosion [16].

All parts of *J. curcas* have also been used in traditional medicine and for veterinary purposes [17]. The leaves and latex are used in healing of wounds, refractory ulcers, and septic gums, and styptic in cuts and bruises [18]. *J. curcas* contains a number of bioactive compounds that are flavonoids, saponins, tannins, and polivenol. The compounds play a role in antibacterial [19], antioxidant, anti-inflammatory, anticancer [20], antidiabetic activities [21], and wound healing [22]. However, limited information is available on the wound healing process of this plant, especially angiogenesis activity.

The aim of this research was to determine the angiogenesis activity of *J. curcas* latex in cream formulation on CD34 immune expression during wound healing phase in mice skin. The results will then be compared to that of treatment with sulfadiazine in standard wound therapy.

## Materials and Methods

### Ethical approval

This research was performed appropriately following the regulation of Animal Ethics Committee. This research was approved by the Animal Ethics Committee of Faculty of Veterinary Medicine, Syiah Kuala University, Banda Aceh, Indonesia (Approval No. 004/KEPH-C/VII/2017).

### Mice

Amount of 36 heads male mice *(Mus musculus)* aged 2 months old and weighing 30-40 g were used in this study. All mice maintained individually in cages at the Laboratory of Pathology, Faculty of Veterinary Medicine, Syiah Kuala University. Feed and water were given *ad libitum* for 2 weeks.

### Preparation of o/w cream formulation

The latex used in the present study acquired a break of *J. curcas* leaf stems which were procured from a local farm around yard areas of Banda Aceh. The latex was collected in the morning with a break of leaf stems, latex at capacity into a sterile bottle. The latex was formulated in cream following the procedure as described by Muntiaha *et al*. [23]. *J. curcas* latex cream made with a base of oil in water (O/W) because the formulation is homogeneity, spreadability, simply to use, non-sticky, soothing on the skin, and easy to clean. A cream base was added little by little with the 10% and 15% latex of *J. curcas* in a porcelain dish containing 100 g of cream and stirred until homogeneous at room temperature. The dosages of 10% and 15% latex of *J. curcas* have been proven to be effective to use for topical for topical wound healing [24].

### Formation of excision wound model

The mice skin wound was done by mean of surgical procedures as explained by Salim *et al*. [24]. Mice were locally anesthetized by topical cream with containing lidocaine 25 mg and prilocaine 25 mg. Longitudinal wounds of about 2.0 cm were made on the paravertebral region till to subcutaneous under the aseptic condition and were observed throughout the experimental study.

### Grouping of mice for experimental design

The mice were separated into four groups contained nine mice of each. group A as a negative control, the mice skin wound received base cream. group B as a positive control, the mice skin wound received standard drug containing sulfadiazine 0.1% cream. group C, the mice skin wound treated with a cream containing *J. curcas* latex 10%. group D the mice skin wound treated with a cream containing *J. curcas* latex 15%. The topical experimentally applied twice daily at 08.00 am and 18.00 pm starting from the day of wounding till to measure on 3^rd^, 7^th^, and 10^th^ post-wounding day.

### Histopathological and immunohistochemical expression of CD34

Wound skin tissue samples were collected in 10% buffered formalin for histopathological examination [25,26]. The tissues were processed by routine paraffin embedding technique, and 5 µm section was stained with immunohistochemical staining by standard methods as described by Darmawi *et al*. [27], using streptavidin-biotin complex. For angiogenesis immunoreaction, we used the CD34 monoclonal mouse antibody (Dako, 1:50 dilution) diluted in 1% BSA in PBS. We defined the scores of CD34 expression as negative, 0 points (Grade 0), mild 1 point (Grade 1), moderate 2 points (Grade 2), and severe 3 points (Grade 3) [28]. The intensity of staining was recorded as weak, moderate, and strong [29].

## Results

**Figure-1:**
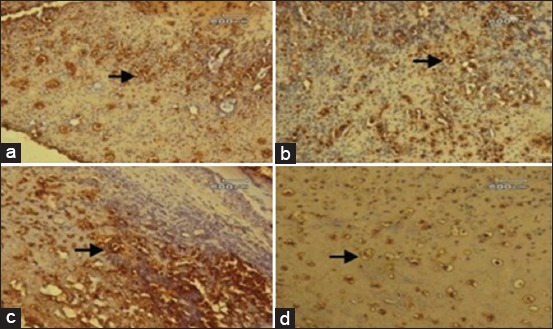
Photomicrograph of wounds skin on day 3 of treatment (streptavidin-biotin 40×). CD34-positive cells in endothelial cells each group that marked the brown color (arrow). (a) Minimal immunoreactivity CD34-positive cells on Group A (Grade 1). (b-d) Moderate immunoreactivity CD34-positive cells on Groups B, C, and D (Grade 2).

**Figure-2:**
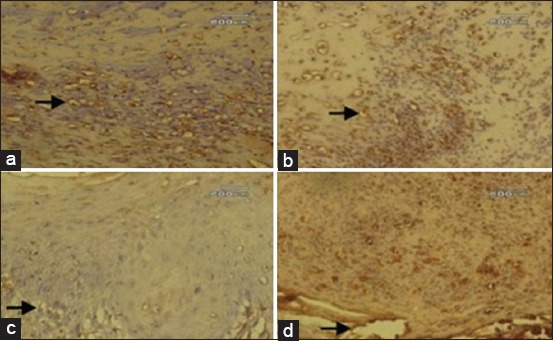
Photomicrograph of wounds skin on day 7 of treatment (streptavidin-biotin 40×). CD34-positive cells in endothelial cells each group that marked the brown color (arrow). (a) Minimal immunoreactivity CD34-positive cells on Group A (Grade 1). (b-d) Moderate immunoreactivity CD34-positive cells on Groups B, C, and D (Grade 2).

**Figure-3:**
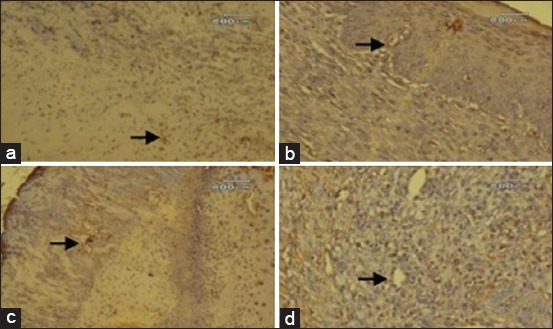
Photomicrograph of wounds skin on day 10 of treatment (streptavidin-biotin 40×). Immunoreactivity CD34 cells each group that marked the brown color (arrow). (a) Minimal immunoreactivity CD34-positive cells on Group A (Grade 1). (b-d) Negative immunoreactivity CD34 cells on Groups B, C, and D (Grade 0).

The results of the present study showed that immunohistochemical of CD34-positive expression is characterized by brown color on microscopic slides. The CD34 monoclonal mouse antibody had a strong reaction with antigen on the endothelial progenitor cells and endothelial cells as shown in Figures-1-3. On day 3, the CD34 positive cells were seen in endothelial cells of each group. The minimal immunoreactivity of CD34 positive cells was observed on group A (Grade 1). Meanwhile the moderate immunoreactivity of CD34 positive cells was found in group B, C, and D (Grade 2) as seen in Figure-1. In this phase, CD34-positive immunoreaction is a sign of angiogenesis or new capillary formation and the migration of endothelial progenitor cells from the vascular.

The results of the day 7 showed that the migration of endothelial progenitor cells and new capillaries more clearly seen. As seen in Figure-2, the minimal immunoreactivity of CD34 positive cells in endothelial cells were observed on group A (Grade 1), but on group B, C, and D we found the moderate immunoreactivity of CD34 positive cells (Grade 2). The cream from 10% and 15% latex of *J. curcas* and sulfadiazine 0.1% cream revealed moderate immune reaction to CD34 on days 3 and 7 in mice skin wound healing.

The results show that the number of endothelial progenitor cells in migratory and differentiated tissue decreases with the endothelial formation on day 10. As shown in Figure-3, the minimal immunoreactivity of CD34 positive cells was only found on group A (Grade 1). However, the negative immunoreactivity of CD34 cell was found on group B, C, and D (Grade 0). In this phase, CD34 is a negative immune reaction in Groups B, C, and D as a sign of the wound healing process.

## Discussion

The simplest interpretation of our finding on day 3 is shown in Figure-1 and day 7 is shown in Figure-2; it was found that the angiogenesis on Groups B, C, and D (Grade 2) is better than the Group A (Grade 1). Guo *et al*. [7] reported a close correlation between an increase in circulating CD34^+^ cells in response to traumatic injury and angiogenesis in traumatic brain injury. A significant increase in the number of circulating CD34+ cells detected in traumatic brain injury. Indeed, the high levels of CD34+ were also observed in 7 days after the injury. The results found that angiogenesis involved in inflammation phase (day 3) and proliferative phase (day 7). The levels of angiogenesis in wounds often correlate with the inflammatory response, largely because inflammatory cells produce an abundance of proangiogenic mediators [7]. In confirmation of our previous study, we found that the cream from the 10% and 15% latex of *J. curcas* revealed moderate immune reaction to CD68 on wound healing [24]. Macrophages as inflammatory cells secrete vascular endothelial growth factor (VEGF) that induces angiogenesis [30].

Here, we described that the CD34 monoclonal mouse antibody had a strong reaction with antigen on the endothelial progenitor cells and endothelial cells. Clinical and animal studies provide evidence that stem progenitor cells are critical for neovascularization [31]. Angiogenesis is the growth of new blood vessels from existing endothelial cells of the wound’s adjacent by mature stromal cells such as fibroblasts [32]. The similar phenomenon described by Ieronimakis *et al*. [33] who noticed that the fraction of CD34^+^ cells remains steady during homeostasis and injury regeneration.

In this study, we agree with and support those of Kirby *et al*. [34] who explained that most hematopoietic stem cells express CD45, and subset of hematopoietic stem cells express CD34. Blood vessels are composed of epithelial tubes with epithelial cells that transport crucial fluid. During lumen formation, endothelial cells in mature blood vessels represented as the first phenotype and second phenotype, and endothelial cells polarization often starts with the delivery through exocytosis, such as CD34 [35].

The effect of *J. curcas* especially on wound healing has been studied using animal models. Many previous reports exist about the efficacy of *J. curcas* on wound healing. Jasmadi *et al*. [36] evaluated that the ointment from 10% latex of *J. curcas* has the potential to accelerate the wound healing of burns on the skin of mice. Salim *et al*. [37] indicated that methanol extract of *J. curcas* leaves was potential in accelerating wound healing in mice. Our previous study indicated that ointment from 10% latex of *J. curcas* has proven to accelerate the epithelial period [38,39], and remodeling period on wound healing in mice skin [40]. This causes that the ointment from 10% latex of *J. curcas* effective to be used for topical therapy on wound healing in mice skin. The quercetine and routine flavonoids at *J. curcas* can improve the wound healing process on the regulation of VEGF expression for the growth of new blood vessels (angiogenesis) [18]. Angiogenesis involved in the proliferative phase which is a principal step in epithelialization of wound healing [5].

This results study showed that on day 10, CD34 is undetected on Groups B, C, and D (Grade 0). The decrease in the number of migratory and differentiated endothelial progenitor cells was in line with endothelial cells formation [41]. DiPietro [42] stated that after an injury, levels of proangiogenic factors increase, reaching a peak slightly before maximum capillary content occurs, and then subside to nearly undetectable levels.

## Conclusion

We concluded that the cream from 10% and 15% latex *J. curcas* has potential as angiogenesis activity in wound healing of mice skin.

## Authors’ Contributions

Darmawi and MNS designed the research; UB and CDI performed immunohistochemistry; UB, Darmawi, CDI, and MNS prepared the manuscript. All authors read and approved the final manuscript.
